# Enhancement of ELDA Tracker Based on CNN Features and Adaptive Model Update

**DOI:** 10.3390/s16040545

**Published:** 2016-04-15

**Authors:** Changxin Gao, Huizhang Shi, Jin-Gang Yu, Nong Sang

**Affiliations:** 1 National Key Laboratory of Science and Technology on Multispectral Information Processing, School of Automation, Huazhong University of Science and Technology, Wuhan 430074, China; cgao@hust.edu.cn (C.G.); shihz_fy@hust.edu.cn (H.S.); nsang@hust.edu.cn (N.S.); 2 Department of Computer Science and Engineering, University of Nebraska-Lincoln, Lincoln, NE 68503, USA

**Keywords:** visual tracking, exemplar-based detection, convolutional neural network (CNN) features, Gaussian mixture model

## Abstract

Appearance representation and the observation model are the most important components in designing a robust visual tracking algorithm for video-based sensors. Additionally, the exemplar-based linear discriminant analysis (ELDA) model has shown good performance in object tracking. Based on that, we improve the ELDA tracking algorithm by deep convolutional neural network (CNN) features and adaptive model update. Deep CNN features have been successfully used in various computer vision tasks. Extracting CNN features on all of the candidate windows is time consuming. To address this problem, a two-step CNN feature extraction method is proposed by separately computing convolutional layers and fully-connected layers. Due to the strong discriminative ability of CNN features and the exemplar-based model, we update both object and background models to improve their adaptivity and to deal with the tradeoff between discriminative ability and adaptivity. An object updating method is proposed to select the “good” models (detectors), which are quite discriminative and uncorrelated to other selected models. Meanwhile, we build the background model as a Gaussian mixture model (GMM) to adapt to complex scenes, which is initialized offline and updated online. The proposed tracker is evaluated on a benchmark dataset of 50 video sequences with various challenges. It achieves the best overall performance among the compared state-of-the-art trackers, which demonstrates the effectiveness and robustness of our tracking algorithm.

## 1. Introduction

Visual tracking is a critical technique to many applications [1,2,3], such as surveillance [4,5], robot vision [6], *etc.* Recently, tracking-by-detection has become an attractive tracking approach [7], which treats tracking as a category detection problem and trains a detector to separate the object from the background. In this class of tracking methods, appearance representation and the observation model (classifier) play important roles, as in detection. Tracking-by-detection methods can be roughly classified into two categories, the generative model and the discriminative model. The latter builds an observation model by online collecting positive and negative examples from the initial or tracked objects. Positive examples are usually sampled near the object location, which may be slightly different from the true object example. These slight differences will be amplified during tracking, leading to further drifting [8].

To address the tracking drift problem with discriminative models, in our previous work, we proposed an exemplar-based model, referred to as exemplar-based linear discriminant analysis (ELDA) [9], which considers tracking as a specific object instance detection task, rather than a general object category detection task as in object detection. Noticing that the trade-off between discriminative ability and adaptivity is crucial to the training of a model-free tracker, we mainly considered the following two aspects in the ELDA framework:

- The exemplar-based model is quite discriminative and specific, because it trains a linear discriminant analysis (LDA) classifier using one positive example and massive negative examples. Besides, to improve its discriminative ability, the ELDA tracker applies histograms of oriented gradient (HoG) features [10] as the appearance representation of the object [9].
- On the other hand, the adaptivity of the exemplar-based model is improved by combining an ensemble of detectors. Each detector (object model) is built based on a positive example; thus, the exemplar-based model can be considered as a template-based method. Model (or template) updating is very important to build a robust tracker.

While ELDA has demonstrated good performance in tracking, we observed that it could still be improved in both discriminative ability and adaptivity.

(1) Discriminative ability

HoG is a quite discriminative representation, which is widely used in object detection tasks [10]. It is well known that HoG is an artificial feature, while learned features have dominated in computer vision recently, especially convolutional neural network (CNN) features. CNN features achieve the best performance in most tasks of object recognition [11,12,13], object detection [14,15], scene labeling [16], action recognition [17], image super-resolution [18], *etc.* It has been shown that the CNN features have even stronger discriminative ability than HoG.

Therefore, a natural question is: Can we use CNN features in visual tracking? To the best of our knowledge, few works have considered that so far. There are two reasons in our opinion. First, very large-scale training data are required to train a deep CNN. However, a small quantity of examples, especially positive examples, can be acquired in the tracking procedure. In recent years, some works proved that CNN features can be considered as a general representation [13]. If the features are extracted by a deep network, they could be exploited for various tasks. That means CNN features with a pre-trained deep network using a large-scale dataset can be used as appearance representation in visual tracking. The second reason is that computing CNN features on each sliding window is quite time consuming for tracking. Girshick *et al.* proposed an R-CNN method for object detection by first selecting a small number of region proposals [14]. However, this strategy is not a good solution to tracking-by-detection methods; because the candidate region is just around the object, and the error of selected proposals will be amplified during tracking, resulting in drifting. Thus, CNN features can be used to improve the discriminative ability, while a fast computation method is required for tracking.

(2) Adaptivity

There are two limitations of the ELDA tracker in the terms of adaptivity: (1) ELDA builds a short-term object model using the object models within a fixed time window *TM*. To ensure robustness, *TM* is typically set to a large value, e.g., *TM* = 500 in [9]. However, such a large number of examples is redundant, because the object instances within a short time window are similar. Thus, selecting a small set of “good” object models is a good solution to this problem. (2) The ELDA tracker models the background as a Gaussian model; however, in fact, the background is quite complex in many cases. A single Gaussian distribution is too simple to describe the complex background. A more powerful model is required.

**Figure 1:**
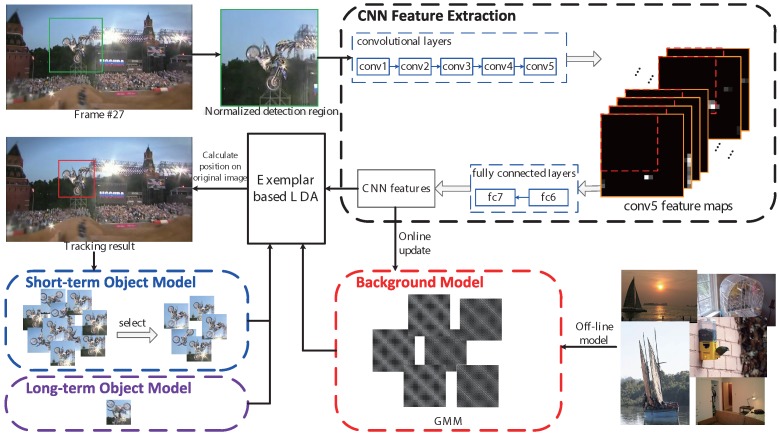
An overview of the ECT tracking algorithm. The convolutional layers and fully-connected layers of convolutional neural network (CNN) features are computed separately. We first compute the Convolutional Layer 5 (*conv5*) feature maps on the whole detection region, and then compute fully-connected layers (*fc7*) on each sliding window. The long-term object model is based on the object instance in the first frame; the short-term object model is a more compact set of the previous object models. The background model is a Gaussian mixture model (GMM), which is initialized offline and updated online. Finally, CNN features are fed to an ensemble of exemplar-based LDA detectors for tracking. The figure is best viewed in color.

To this end, this paper presents an exemplar-based tracker based on CNN features and adaptive model update for visual tracking (ECT), as shown in Figure 1. CNN features are introduced into the exemplar-based tracking method for appearance representation and sped up by separately computing convolutional layers and fully-connected layers. The VGG-F CNN architecture [19] is used in this paper, which contains five convolutional layers (*conv1–5*) and three fully-connected layers (*fc6–8*). Generally, the feature of the seventh fully-connected layer (*fc7*) is used as a representation for many tasks in computer vision. To speed up CNN feature extraction in this architecture, we first compute the fifth convolutional feature maps (*conv5*) on the whole detection region and then compute the seventh fully-connected layer (*fc7*) on each sliding window. To improve the adaptivity, we propose a method to update object models by selecting the detectors with strong discriminative ability and uncorrelated to other selected detectors; and build the background as a Gaussian mixture model (GMM) to cover the complex variations of the scenes.

There are four main contributions: (1) we introduce CNN features into the visual tracking tasks, without training a deep network; (2) we proposed a two-step CNN feature extraction method to speed up the algorithm; (3) a new strategy is proposed to update object models according to discriminative ability and correlation; (4) GMM is used to build the background model, to improve the adaptivity in the complex scene.

The rest of this paper is organized as follows. Related work is reviewed in Section 2. The proposed ECT tracker is introduced in detail in Section 3. In Section 4, experimental results are presented. Additionally, we conclude this paper in Section 5.

## 2. Related Work

### 2.1. Exemplar-Based Tracker

Unlike most tracking-by-detection approaches, ELDA considers visual tracking from a different view, *i.e.*, an object instance detection, rather than an object category detection. It alleviates the drifting problem caused by the error of the samples used to train a classifier. The proposed tracker follows the framework of the ELDA tracker [9]; thus, we first briefly introduce the ELDA tracker. As a tracking-by-detection algorithm, the ELDA tracker uses an ensemble of exemplar-based detectors to distinguish a target object from its local background. Each exemplar-based detector is trained using only one positive sample and massive negative samples, which can be considered as a template-based method. This method is motivated by the work of exemplar-based SVM (ESVM). The HoG feature is used as the appearance representation. That is to say, this work follows the “HoG + ESVM” framework [20], which is quite popular in object detection or mid-level part detection. The LDA classifier can be considered as a fast alternative to the SVM classifier [21].

### 2.2. Appearance Representations in Tracking-By-Detection Methods

Recently, tracking-by-detection has become an attractive tracking technique, which treats tracking as a category detection problem and trains a detector to separate the object from the background. We first refer the readers to some surveys [7,22,23,24]. The reason for the good performance of existing tracking-by-detection methods is that many of them borrow some ideas from the the successful detection methods, both in appearance representations and classifiers. Tracking-by-detection methods can be classified into two categories, the discriminative model and the generative model. We will first review some works along both lines, followed by some classifier techniques.

#### 2.2.1. Discriminative Models

Haar-like features are among the most commonly-used representations in tracking [8,25,26,27,28,29], especially in discriminative models. That is motivated by a popular detection method [30], which combines Haar features and a boosting classifier together for detection. The most successful application of this method is face detection. The binary pattern is another common representation method in detection, which has also been introduced into tracking [31,32]. Ma and Liu used compact binary code to represent object appearance with hashing techniques [33].

The Haar-like feature and binary pattern are very simple and fast; however, the discriminative ability is not good enough for some variations of the objects. Some more powerful features are used in tracking, for example the HoG feature [9,34,35,36,37] and the scale-invariant feature transform (SIFT) feature [38].

Part-based models are also widely used in detection, due to their robustness to deformations, occlusions, *etc.*, which, naturally, have been borrowed to design a tracker [39].

#### 2.2.2. Generative Model

The generative model-based methods are another branch of detection methods. This is also widely used in tracking [40], by modeling the generative process of object/background and detecting the most similar candidate in video sequences. The representation methods in this category, such as PCA [41], sparse coding [42,43,44,45,46,47] and sparse PCA [48,49], are incorporated into tracking-by-detection algorithms.

Besides, some works combine tracking (-by-detection) and segmentation together for highly non-rigid object tracking [50,51,52]. These methods perform well, especially with some challenging attributes, such as deformations, occlusions, rotation and scale changes, by using the results of segmentation to refine the tracking (-by-detection) model.

### 2.3. LDA

As a fundamental data analysis method that can be widely used for classification, dimensionality reduction, interpretation of the importance of the given features, *etc.*, LDA has been widely used in computer vision, as well as other fields. However, LDA has several limitations in practice, and many studies focus on these problems. For example, LDA can only be used in the linear case, which however can be possibly extended to nonlinear LDA using the kernel trick [53]; the variance matrix may be singular, called the singularity problem, which can be solved by using PCA as a pre-processing step and then performing LDA [54] or using a representation model that represents a sample as a matrix rather than a vector and the collection of data as a collection of matrices than a single large matrix accordingly [55]; the original version of LDA is for two classes, and Rao extended LDA to multi-class cases [56], to find a subspace that appears to contain all of the class variability. In our paper, the exemplar LDA model [21] is used to train a detector of each object exemplar. Exemplar LDA can be regarded as a variant of LDA in the particular case of exemplar-based settings. Therefore, theoretically speaking, it is likely that ELDA will be subject to the general limitations encountered in using LDA. That is the reason we model the background as a GMM to adapt to the complex scenes, which is quite similar to multiple class LDA.

### 2.4. Deep Networks in Tracking

While deep networks have been successfully used in many computer vision tasks, such as image classification, object recognition, object detection, action recognition, segmentation, *etc.*, they are not so popular in object tracking, probably due to two reasons, that is the lack of training data and the high computational complexity. However, some attempts have been made in this filed in recent years. To address the problem of the lack of labeled samples, the authors in [57] and [58] trained a specific feature extractor offline using a large number of auxiliary data, with convolutional neural networks and stacked denoising autoencoder networks, respectively. Zhang *et al.* attempted to resolve this problem in a different way, by extracting CNN features feed-forward for object tracking without learning, which means no auxiliary data are required. To simultaneously deal with the two aforementioned problems, Li *et al.* proposed the DeepTrack method, which learns feature representation online using a single CNN. In this paper, we use CNN features as a representation, for which the network is trained offline. However, CNN in our work is general, which means, unlike [57] and [58], we do not need to train a specific network for our tracking algorithm. Although these deep learning-based tracking methods have been proposed, we believe that more natural and effective ways to use deep networks in tracking remain to be further explored.

### 2.5. Differences with ELDA

As mentioned above, our work follows the framework of ELDA proposed in [9], which uses an ensemble of ELDA detectors for tracking. Our method has four main differences with ELDA in terms of three modules (as presented in [7]) in tracking:

(1) Representation scheme. The proposed method uses CNN features for object representation, while ELDA uses HoG features. Many recent works have proven to have better performance of CNN features in object detection and many other computer vision tasks;
(2) Search mechanism. Both methods adopt the dense sampling search mechanism. However, our method samples the candidate windows on the *conv5* feature maps; while ELDA samples the windows on the original image. The step length of the sliding window of the proposed method, corresponding to the ordinal image, is not a fixed value, as seen in Section 3.2;
(3) Object model update. ELDA selects all of the models in a fixed time window to build the short-term object models; while our method selects a small number of models by considering their discriminative abilities and correlation among them. The models in our method are more compact.
(4) Background model. The ELDA tracker builds the background model as a single Gaussian model; while the proposed method builds it as a Gaussian mixture model to improve its adaptivity in complex scenes.

## 3. Our Method

### 3.1. ELDA Tracker

The ELDA tracker in frame *k* is composed of an ensemble of ELDA detectors:

(1) $$H_{k}\left(X\right)=\lambda_{1}H_{1}\left(X\right)+\sum_{i=2}^{k}\lambda_{i}H_{i}\left(X\right)$$

where *X* denotes a feature vector of a candidate sub-window and $H_{k}\left(X\right)$ denotes an ELDA detector obtained in frame *k*, which can be written as:

(2) $$H_{k}\left(X\right)=\omega_{k}^{T}\times X$$

(3) $$\omega_{k}=\Sigma_{k}^{-1}\left(X_{k}^{p}-\mu_{k}\right)$$

where the value of $H_{k}\left(X\right)$ is the confidence score corresponding to *X*, $\omega_{k}$ denotes an object model and $\left(\Sigma_{k},\mu_{k}\right)$ is the background model.

ELDA consists of four parts: the long-term object model, the short-term object model, the offline background model and the online background model. For more details of them, we refer the readers to [9]. This work follows exactly the same framework as [9] in building all of the components except for the short-term object model.

This section mainly focuses on the improved parts of our method. First, we use CNN features (fast method) as appearance representation rather than HoG, presented in Section 3.2. CNN features are proven to be more discriminative. Second, we update the short-term object model by a new strategy, as seen in Section 3.3. Thus, a small number of effective object models is used for tracking. Finally, we model the background as a GMM, and update it online, introduced in Section 3.4.

### 3.2. Appearance Representations

We introduce CNN features for appearance representation into the exemplar-based tracker, which represents an image region of a searching window as a holistic descriptor. We follow the “CNN + SVM (LDA)” framework to build the exemplar based detectors. That is to say, the 4096-dimensional CNN feature vector of a positive sample is fed into an exemplar LDA classifier to train a detector. Since the input to CNN is of the fixed size *N* × *N* × 3 (224 × 224 × 3 in the VGG-F CNN architecture [19]), we normalize the windows of the size *w* × *h* × 3 (*w* and *h* are the width and height of the windows, respectively) to *N* × *N* × 3 by bilinear interpolation, to fit the CNN network. The 4096-dimensional feature vector is usually taken on the sixth or seventh fully-connected layer (*fc6* or *fc7*).

Calculating the 4096-dimensional feature vector over each densely-sampled window directly is quite time consuming, because some convolutional features are computed several times. We notice that the convolutional layers can generate the feature maps of any sizes, in a sliding window manner, and the convolutional layers need not to have a fixed image size; while the fully-connected layers require a fixed feature (input) size. That is to say, the fixed size constraint comes only from the fully-connected layers [12]. Hence, to speed up the computation of the CNN features of all of the candidate sliding windows, we separately operate the convolutional layers and the fully-connected layers in two steps, as shown in Figure 1. This is partly motivated by [12]. Specifically, the algorithm of two-step CNN features extraction is shown in Algorithm 1.
**Algorithm 1** Two-step CNN feature extraction**Input:**
The image region to be detected;**Output:**
The CNN features of each sub-window;The bounding box of tracking result in the original image;1:pre-compute the *conv5* feature maps on the entire detection region;  2:slide the 13 × 13 window on *conv5* feature maps, and compute *fc7* features on these patches;  3:compute the scores of the patches using the ECT tracker, and find the final result by the non-maximum suppression (NMS) algorithm;  4:calculate the bounding box (tracking result) in the original image corresponding to each window on the *conv5* feature maps by Equations (4) and (5).


It can be seen that we compute the *conv5* feature maps over the entire region only once, which avoids repeatedly computing the convolutional features. The candidate patches for detection are densely sampled on the *conv5* feature maps. In the VGG-F CNN architecture [19], the size of candidate patches is 13 × 13, which fits the input size of *fc6*. For each candidate patch, its representation is the output feature of *fc7*. Next, we introduce the method of position transition, from *conv5* feature maps to the normalized image and from the normalized image to the original image. We denote a region position *P* in an image or a feature map using the top-left position $p_{l,t}$ and bottom-right position $p_{r,b}$. The position of a result window in a *conv5* feature map is denoted as $P^{conv5}$. Then, we present how to obtain the final position $P^{ori}$ in the original image. The corresponding position in the normalized image region $P^{nor}$ can be calculated by:

(4) $$\left\{\begin{array}{c} p_{l,t}^{nor}=\left(p_{l,t}^{conv5}-1\right)\times s+\left\lfloor \gamma \right\rfloor -o_{l,t}\\ p_{r,b}^{nor}=\left(p_{r,b}^{conv5}-1\right)\times s-\left\lceil \gamma \right\rceil +o_{r,b} \end{array}\right.$$

where *γ* is the radius of the receptive field in the normalized image of each pixel in *conv5* feature maps, *s* is the effective stride and $o_{l,t}$ and $o_{r,b}$ are respectively the offsets of the top-left and bottom-right positions, as in [12]. Additionally, the position $P^{ori}$ in the original image can be obtained by:

(5) $$\left\{\begin{array}{c} p_{l,t}^{ori}=p_{l,t}^{nor}\times w/N\\ p_{r,b}^{ori}=p_{r,b}^{nor}\times h/N \end{array}\right.$$

**Figure 2:**
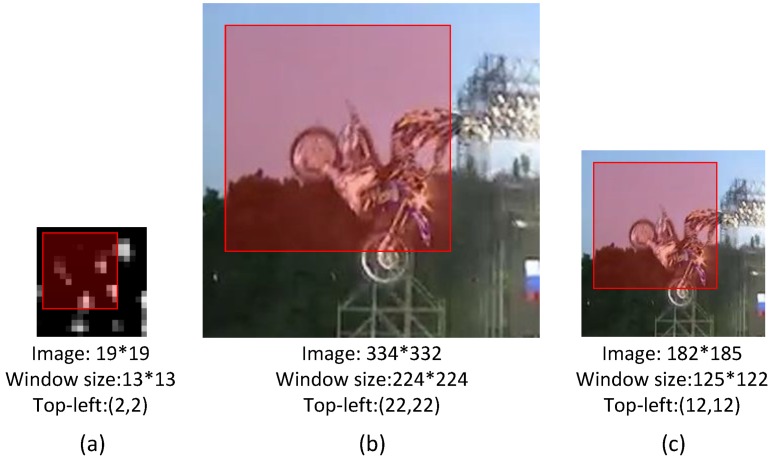
Corresponding relations of positions in: (**a**) *conv5* feature maps; (**b**) the normalized image region; and (**c**) the original image. The red rectangles denote the corresponding regions in these three images. The positions of the windows (with top-left coordinates and sizes) in different images are listed below these images.

We present an example to illustrate how to calculate the final position in the original image in Figure 2. Note that, in our work, the step length of the sliding window may vary with the size of the object window for different video sequences, which can be approximated by:

(6) $$\left\{\begin{array}{c} S_{hor}=round\left(16\times w/N\right)\\ S_{ver}=round\left(16\times h/N\right) \end{array}\right.$$

where $S_{hor}$ and $S_{ver}$ are the horizontal and vertical step lengths, respectively.

### 3.3. Object Model Update

The exemplar-based model with CNN features is quite discriminative. To build a robust tracker, we should improve its adaptivity by updating short-term object models (note that, the way to build the long-term object model is the same with [9]). In this paper, the short-term object model is updated by selecting the detectors with strong discriminative ability and uncorrelated to other selected detectors. Gao *et al.* employed a sliding time window to select the object models [9]. Some other methods can be used, as well, like cluster methods, the random selection method, and so forth. For the precision and running speed of our tracker, we propose a greedy method to select “good” object models, as shown in Algorithm 2. We first rank all of the short-term object models by their discriminative ability, resulting in a candidate set of object models. We then take an object model from the candidate set in order and add it into the final short-term object model set, if it is uncorrelated to other selected detectors. In our method, the discriminative ability of a model $\omega_{i}$ is measured by the confidence score $H_{i}\left(X_{k}\right)$ to the current positive example $X_{k}$, while the correlation between a candidate model $\omega_{u}$ and a selected model $\omega_{z}$ is measured by the distance of their confidence scores to all tracking results. More precisely, the distance $dis_{z}$ from $\omega_{u}$ to $\omega_{z}$ can be computed as:

(7) $$dis_{z}=\sum_{i=1}^{k}{\left(H_{u}\left(X_{i}\right)-H_{z}\left(X_{i}\right)\right)}^{2}$$

Note that, the model size *Z* < *L* is possible, in which case we can change the threshold *τ* to repeat our algorithm. However, considering the efficiency of our method, we will not do this more than once.
**Algorithm 2** Object model updating algorithm**Input:**
The set of all the short-term object models $\left\{\omega_{i}\right\}$, (i=2,3,…,k);The set of the representations $\left\{X_{k}\right\}$ of tracking results; *k* is the index of current frame;The predefined threshold *τ*;The maximum size of final object models *L*;**Output:**
The final object models R;1:rank all the models $\left\{\omega_{i}\right\}$ using the scores of $H_{i}\left(X_{k}\right)=\omega_{i}^{T}\times X_{k}$;2:take the top *M* models as candidates $C=\{\omega_{m}\}$, m=1,2,…,M;3:take the first model $\omega_{u}$ (*u* = 1) from C, and put it into R; its size is *Z* = 1;4:**while** *Z* ≤ *L&u* ≤ *M* **do**5:  take a model from C in order (from two to *M*), denote as $\omega_{u}$;6:  *flag* = 1;7:  **for** *Z* = 1 to *Z* **do**8:      compute the distance $dis_{z}$ from $\omega_{u}$ to $\omega_{z}$ by Equation (7);9:      **if** $dis_{z}<\tau$ **then**10:            *flag* = 0;11:             break;12:      **end** **if**13:  **end** **for**14:  **if** *flag* == 1 **then**15:      put $\omega_{u}$ into R;16:      *Z* = *Z* +1;17:  **end** **if**18:**end while**


### 3.4. Background Model Update

We model the background as a GMM with *C* components, denoted by:

(8) $$M^{B}=\left\{\left(p_{c},\mu_{c},\Sigma_{c}\right)\right\},c=1,2,...,C$$

where $p_{c}$, $\mu_{c}$, $\Sigma_{c}$ respectively represent the prior probability, mean and covariance of the *c*-th component. Let us denote by $M_{k}^{B}={\left\{\left(p_{k,c},\mu_{k,c},\Sigma_{k,c}\right)\right\}}_{c=1}^{C}$ the background model maintained at the time instance *k*. The initial model $M_{0}^{B}$ is built offline, by using a large amount of negative samples on some natural images. We use the expectation maximum (EM) algorithm to calculate $M_{0}^{B}$.

We update the background model online to improve the adaptivity by some negative samples quite relevant to the tracking task. At the time instance *k*, we calculate the model $M_{k}^{B}$ using the previous model $M_{k-1}^{B}$ and new negative samples. Let us denote by $X_{k,c}$ the collection of the $n_{c}$ negative samples that match the *c*-th component and by $N_{k,c}$ the number of samples used in calculating $M_{k}^{B}$, and let $S_{k,c}=X_{k,c}\cdot X_{k,c}^{T}$. The iteration step of the online background model update can then be given by:

(9) $$p_{k,c}=p_{k-1,c}+\alpha \left(1-p_{k-1,c}\right)$$

(10) $$N_{k,c}=N_{k-1,c}+n_{c}$$

(11) $$S_{k,c}=S_{k-1,c}+X_{k,c}\cdot X_{k,c}^{T}$$

(12) $$\mu_{k,c}=\mu_{k-1,c}*N_{k-1,c}/N_{k,c}+\mu_{k,c}$$

(13) $$\Sigma_{k,c}=\left(S_{k,c}-N_{k,c}\mu_{k,c}\cdot \mu_{k,c}^{T}\right)/\left(N_{k,c}-1\right)$$

where *α* is the learning rate.

## 4. Experimental Results

In this section, we evaluate the proposed tracking method (denoted as ECT), in comparison with other state-of-the-art trackers, on the CVPR2013 benchmark dataset [7] consisting of 50 sequences. This dataset covers 11 challenging scenarios for visual tracking, *i.e.*, illumination variation, scale variation, occlusion, deformation, motion blur, fast motion, in-plane rotation, out-of-plane rotation, out-of-view, background clutters and low resolution. This dataset is an up-to-date comprehensive benchmark specifically designed for evaluation of tracking performance.

### 4.1. Implementation Details

For the object representation, we extract CNN features for appearance representation in this work, using the public MATLAB code *MatConvNet* (the code of MatConvNet can be found at: http://www.vlfeat.org/matconvnet/). We apply *imagenet-vgg-f* [19] as the pre-trained CNN model, which was trained to perform object classification on ILSVRC13 [59]. The input image of this CNN architecture is 224 × 224 × 3 in dimension; the *conv5* feature maps are 13 × 13 × 256 in dimension; and the feature vector on *fc7* is 4096 in dimension. In our two-step CNN extraction process, the size of input image is no smaller than 224 × 224; the size of *conv5* feature maps is related to the input image; and the size of sliding windows on *conv5* is 13 × 13, each window products of an *fc7* feature vector of 4096 in dimension. Here are other parameters of this network in Equation (4): the radius of the receptive field *r* = 139/2, the stride *s* = 16, the top-left offset $o_{l,t}=63$ and the bottom-right offset $o_{r,b}=75$ [12]. For the object model updating, the size of candidate models is *M* = 100; the maximum size of the final models is *L* = 20; and the threshold is *τ* = 0.3. For the background model updating, the number of Gaussian distributions in GMM is *C* = 7; the learning rate is *α* = 0.7.

To build the offline background model, we collected more than 1,000,000 patches (64 × 64 pixels) by randomly sampling on the 5096 images of the PASCAL VOC 2008 dataset [60]. Then, the CNN feature is extracted to build the initial background model by normalizing them to 224 × 224 using bilinear interpolation; and the online negatives are sampled in the ring area with 5 < *d* ≤ 30. The detecting area $R_{d}$ is also set to 30. Note that the step of the sliding window relates to the size of object. This sample scheme is coarser than the dense sample scheme in general.

The tracker was implemented using MATLAB and C/C++. The average time cost for all testing sequences is about 2 fps on a workstation with Intel Xeon E5-2650 CPU (2.6 GHz) and an Nvidia Tesla K20C GPU. In our implementation, CNN features and the exemplar LDA classifier are computed using GPU. We also test the average time cost with the CPU only, which is about 0.6 fps. Note that our code is implemented without intensive program optimizations, such as parallel programming, which can be used to reduce the time cost of our tracker.

### 4.2. Overall Performance

We run the one-pass evaluation (OPE) [7] on the CVPR2013 benchmark using the proposed ECT tracker. Many trackers are compared in our experiment. The work [7] compares 29 popular trackers on this benchmark dataset. Besides the 29 trackers, we also compare our tracker to some other trackers, whose results on the benchmark are reported in recent past, *i.e.*, SCEBT [61], ELDA [9], KCF [36], TGPR [62] and DLT [58]. Thus, 35 trackers in total are compared in our experiment.

Two common evaluation criteria are used for quantitative comparison, namely the precision plot and the success plot, proposed by [7]. First, we define these two criteria briefly. Both of them measure the percentage of successfully-tracked frames over an entire video against the densely-sampled threshold. In the precision plot, the threshold is the central location error (*CLE*), while in the success plot, the threshold is the bounding box overlap ratio (*OR*). For each frame, the result is denoted as the tracked bounding box $B_{T}$ and the central location $C_{T}$, which for the ground truth are $B_{G}$ and $C_{G}$, respectively. *CLE* is defined as the average Euclidean distance (in pixels) between $C_{T}$ and $C_{G}$. *OR* is defined by the intersection over union (IOU) metric $\frac{area(B_{T}⋂B_{G})}{area(B_{T}⋃B_{G})}$. To rank the trackers, we use the threshold metric (at 20) for the precision plot, while the area under the curve (AUC) metric for the success plot. The evaluation plots are computed using the tool provided by [7].

**Figure 3:**
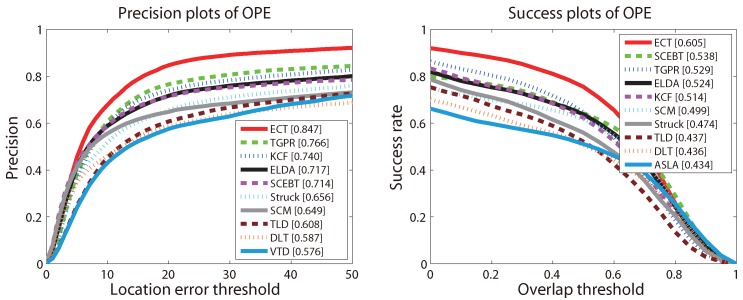
The precision plot and success plot for one-pass evaluation (OPE) on the CVPR2013 benchmark. The top ten trackers with respect to the ranking scores are shown in each plot. The figure is best viewed in color.

The precision and success plots for OPE are shown in Figure 3. All 35 trackers mentioned above are compared in this experiment, but only the top ten trackers with respect to the ranking scores are reported in each plot. The trackers appearing in these two plots and not mentioned above are as follows: Struck [29], SCM [44], TLD [31], VTD [49], ASLA [43]. The ranks are set with the score at threshold 20 and the AUC score for precision and success plots, respectively. The scores are also presented in the square brackets with the name of the trackers. Figure 3 shows that the proposed ECT achieves overall the best performance using both metrics. The remarkable performance gain obtained on such a large dataset demonstrates that our proposed method is very robust to the general challenges in tracking (the detailed results of the proposed method can be found on the project web page: https://sites.google.com/site/changxingao/ecnn).

**Figure 4:**
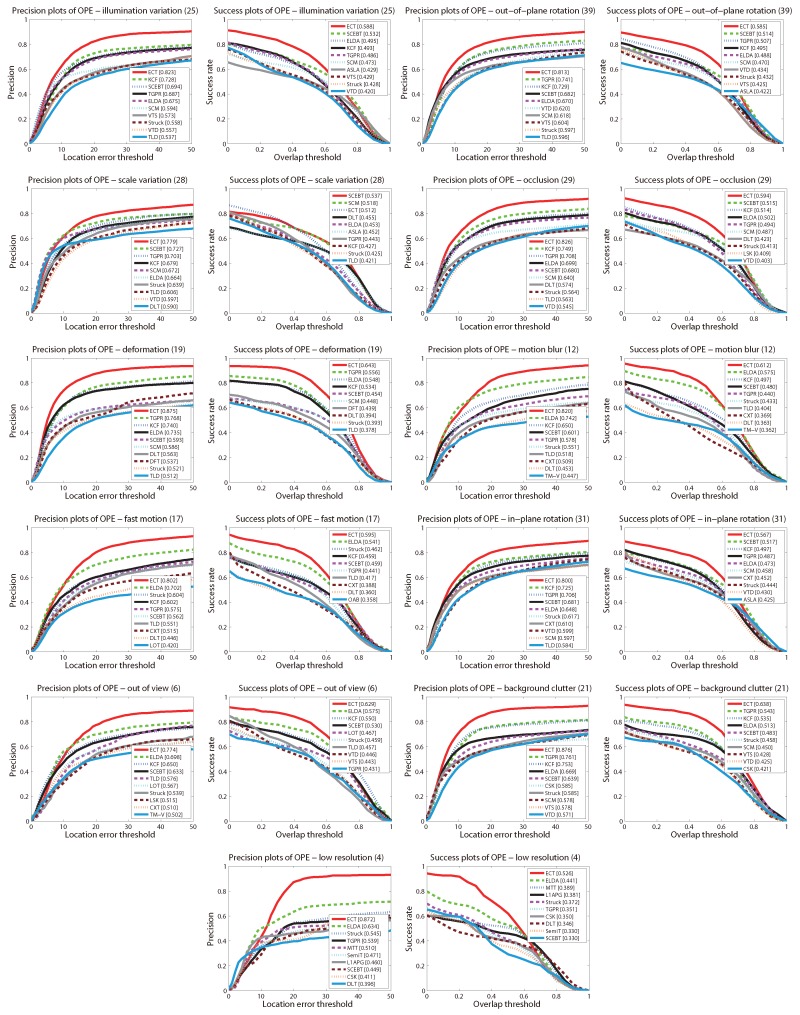
Average precision plot and success plot for OPE on the tracking benchmark dataset with 11 challenging attributions. The top ten trackers with respect to the ranking scores are shown in each plot. The figure is best viewed in color.

Then, we discuss the performance of ECT with the 11 challenging attributes, the precision and success plots are shown in Figure 4. The ranks are set with the score at threshold 20 and the AUC score for the precision and success plots respectively. It can be seen that ECT achieves the best performance in all 11 attributes using both metrics, expect in scale variation using the success plot. That demonstrates the robustness of our tracker to various challenges, especially to deformations, fast motions, background clutters and low resolution challenges. As mentioned above, ECT dose not rank first with scale variation challenge using the success plot. We believe the reason is that the detectors in ECT are designed for a fixed scale.

### 4.3. Quantitative Comparison

To further evaluate the robustness of our method in detail, we compare it against the other seven trackers, the top two of the 29 trackers reported in [7] (Struck [29], SCM [44]) and five trackers mentioned above (SCEBT [61], KCF [36], TGPR [62], DLT [58], ELDA [9]). For better analysis of the effectiveness of the ECT tracker, we first report the tracking performance at several different thresholds based on both the precision metric and the success rate metric in Table 1 and Table 2, respectively. The ECT tracker consistently outperforms other trackers at different thresholds. These comparison results demonstrate the superiority of the ECT tracker.

**Figure 5:**
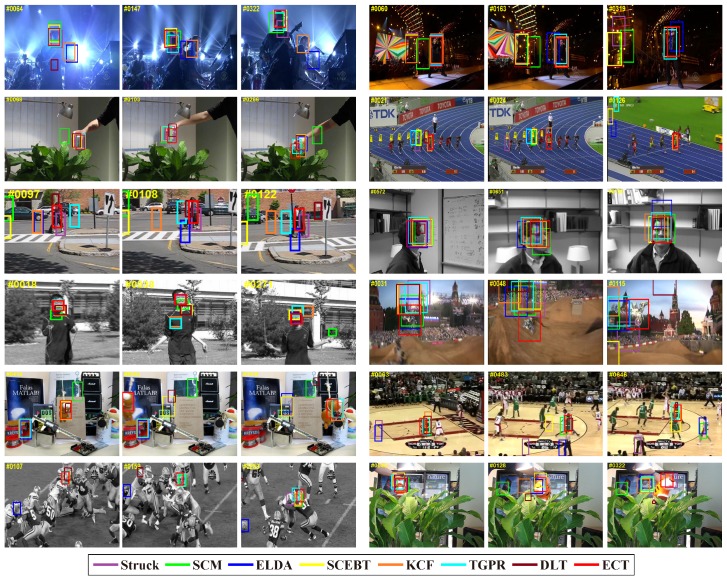
Tracked bounding box results comparisons of eight trackers in 12 videos. The figure is best viewed in color.

For intuitive demonstration, Figure 5 presents a qualitative comparison of the tracked bounding box with the eight trackers using both metrics on twelve challenging sequences. The challenging attributes of these sequences have been annotated in the benchmark [7]. The comparison results demonstrate the good performance of the ECT tracker in both accuracy and adaptivity.

Although ECT does not need to train a deep network during tracking, the CNN features are used to represent objects. Thus, we also compare our method to some deep learning-based tracking algorithms, *i.e.,* DLT [58], DeepTrack [63], CNT [64], SO-DLT [65], in terms of the score at threshold 20 for the precision plot and the AUC score for the success rate plot; because the detailed results of the bounding box at each frame of DeepTrack, CNT and SO-DLT are not public. The comparison results are reported in Table 3, which shows that ECT performs comparably to DeepTrack and significantly better than the others.

### 4.4. Evaluation of Components

To verify the contributions of each of the three proposed components, including the appearance representation using CNN features, the object model and the background model, we build three variants of the ECT tracker for comparative study, which are detailed as below:

- ELDA_CNN: replacing the HoG representation in ELDA with CNN features;
- ECT-OM: removing the proposed object model from ECT;
- ECT-BM: removing the proposed background model from ECT;

Note that, in building these variant trackers, we keep unchanged everything except for the highlighted modifications above. Furthermore, note that we include the ELDA tracker in our comparison, as well, since the current work basically follows the ELDA framework.

**Figure 6:**
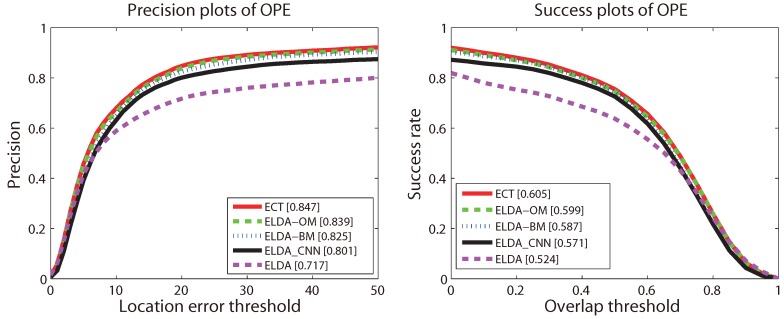
The precision plot and success plot of different versions of our method for OPE on the CVPR2013 benchmark. The top ten trackers with respect to the ranking scores are shown in each plot. The ranks are set with the score at threshold 20 and the AUC score for precision and success plots, respectively. The figure is best viewed in color.

The comparison results are reported in Figure 6. It can be seen that ELDA_CNN obviously outperforms ELDA; this proves that CNN features play the most important role in the ECT tracker. That is because appearance representation is most critical (as pointed out in [66]), and CNN features are quite discriminative. ECT-OM performs better than ECT-BM, which means that the object model is less important than the background model. The reason is that, modeling the background as a GMM allows our tracker to adapt to the complex scenes, while selecting a subset of object instances to build an object model designed in consideration of tracking speed. Surprisingly, ECT slightly outperforms ECT-OM, which means the selected compact object model is more effective, by dropping out some bad models.

## 5. Conclusions

This paper has proposed to enhance the ELDA tracking algorithm by CNN features and adaptive model update. CNN features are used as the object representation; and a two-step CNN feature extraction method has been proposed for fast computation; an object model update method is employed to build a compact object model; and the background model is described using a GMM. Promising results on video sequences of the CVPR2013 benchmark with various challenges showed that our method outperforms the state-of-the-art tracking algorithms, which demonstrated the robustness of our method. We are considering the following for the future work. We are searching for a method to refine the network during the tracking procedure, with low time cost. In this paper, the CNN architecture is pre-trained, which is not sufficiently specific to track an object instance.

## Figures and Tables

**Table 1:** Tracking performance at different thresholds based on the precision metric for 8 trackers on the benchmark dataset (p@*x* means the precision metric at the central location error *x*). Bold and underlined values indicate best and second best performance.

Methods	p@5	p@10	p@15	p@20	p@25
Struck [29]	0.355	0.519	0.605	0.656	0.690
SCM [44]	0.416	0.557	0.617	0.649	0.670
ELDA [9]	0.416	0.589	0.667	0.717	0.744
SCEBT [61]	0.433	0.591	0.673	0.714	0.740
KCF [36]	0.365	0.592	0.697	0.740	0.767
TGPR [62]	0.384	0.607	0.713	0.766	0.791
DLT [58]	0.349	0.461	0.540	0.587	0.613
ECT	**0.457**	**0.679**	**0.788**	**0.847**	**0.876**

**Table 2:** Tracking performance at the different thresholds based on the success rate metric for 8 trackers on the benchmark dataset (p@*x* means the success rate metric at the bounding box overlap ratio *x*). Bold and underlined values indicate best and second best performance.

Methods	p@0.3	p@0.4	p@0.5	p@0.6	p@0.7
Struck [29]	0.669	0.614	0.559	0.476	0.354
SCM [44]	0.681	0.656	0.616	0.548	0.440
ELDA [9]	0.727	0.685	0.637	0.555	0.431
SCEBT [61]	0.738	0.690	0.642	0.581	0.482
KCF [36]	0.730	0.683	0.623	0.524	0.393
TGPR [62]	0.769	0.716	0.646	0.539	0.377
DLT [58]	0.591	0.558	0.507	0.442	0.358
ECT	**0.852**	**0.810**	**0.755**	**0.656**	**0.488**

**Table 3:** Comparison of the deep learning-based trackers and our approach in terms of score at threshold 20 for the precision plot (TP) and the AUC score for the success rate plot (ASR), on the CVPR2013 benchmark. Bold and underlined values indicate best and second best performance.

Methods	DLT	DeepTrack	CNT	SO-DLT	ECT
TP	0.587	0.83	0.612	0.819	**0.847**
ASR	0.436	**0.63**	0.471	0.602	0.605

## Abbreviations

AUC: area under the curve
CNN: convolutional neural networks
ELDA: exemplar-based linear discriminant analysis
HoG: histograms of oriented gradients
GMM: Gaussian mixture model
IOU: intersection over union
LDA: linear discriminant analysis
NMS: non-maximum suppression
OPE: one-pass evaluation
SIFT: scale-invariant feature transform
